# A Text Book of Pathological Anatomy and Pathogenesis

**Published:** 1887-06

**Authors:** 


A TEXT-BOOK OF PATHOLOGICAL ANATOMY AND PATHOGENESIS. *By* ERNEST ZIEGLER. *Translated and edited for English students by* DONALD MACALISTER, *Part II., Special Pathology, Sections ix–xii. London and New York:* MCMILLAN & CO., *Chicago:* W. T. KEENER.

The volume in hand is devoted to the consideration of the anatomico-pathological changes together with the causes producing them in the urinary organs, the respiratory organs, and in the central and peripheral nervous systems. It furnishes the portion, Special Pathology, which, with Part I on General Pathology, completes the work. Together they cover the whole field of Pathology. The generally accepted views at the present time are briefly and clearly stated. Besides the text there are numerous explanatory and supplementary notes setting forth the differing opinions upon the subjects treated of. The value of the work as a hand-book, as well as a text-book, is also greatly enhanced by extensive bibliographical references. The arrangement of the matter in paragraphs with the subjects in heavy faced type adds very much to the convenience in using the book. The publishers' imprint is sufficient guarantee for its typographical excellence.

